# Prevalence and molecular characteristics of colistin-resistant *Escherichia coli* in livestock and fresh meat detected by a selective enrichment method

**DOI:** 10.1007/s11259-026-11463-2

**Published:** 2026-08-26

**Authors:** Camille Fleury, Javier E. Fernandez, Vincent Perreten, Gudrun Overesch

**Affiliations:** Institute of Veterinary Bacteriology, Vetsuisse Faculty, University of Bern, Länggassstrasse 122, Bern, CH-3012 Switzerland

**Keywords:** Antimicrobial resistance, Broilers, Calves, Pigs, Colistin, *mcr* genes

## Abstract

Considering the increase of infections with carbapenemase-producing Enterobacterales in human medicine*,* colistin is nowadays listed as highest priority critically important antimicrobial for human medicine by the World Health Organization, leading to drastic restrictions on its previous widespread use in veterinary medicine. Because of the reduced but continued use of colistin in veterinary medicine, livestock and fresh meat might represent a source of colistin-resistant bacteria. Here, we performed a culture-based study followed by whole genome sequencing (WGS) to detect and characterise colistin-resistant *Escherichia coli* in caecal samples of Swiss livestock and fresh meat using the random sample plan of the European programme on antimicrobial resistance in zoonotic and indicator bacteria from humans, animals and food. A low prevalence of colistin-resistant *E. coli* in Swiss livestock was detected (2.7% in calves (n = 298), 2.9% in pigs (n = 350), 0.6% in broilers (n = 805)). In fresh meat, the prevalence of colistin-resistant *E. coli* was comparable for beef (3.1%, n = 164) and pork (3.7%, n = 163), while chicken meat had a higher prevalence of 5.4% (n = 295). WGS highlighted that colistin resistance was mostly associated with chromosomal mutations (56%), while a lower occurrence of *mcr* genes (22%) was found. WGS analysis revealed a high diversity in the detected colistin-resistant *E. coli* as they belonged to 32 sequence types (ST) and 48 core genome STs. Fresh meat might be contaminated with colistin-resistant *E. coli,* but its detection is not currently implemented in the European programme on antimicrobial resistance. By an approach using selective bacterial culturing all phenotypically colistin-resistant *E. coli* can be detected independently from their molecular resistance mechanisms, with the disadvantage of eventually also inducing chromosomal mutations during selective overnight incubation.

**Supplementary Information:**

The online version contains supplementary material available at https://doi.org/10.1007/s11259-026-11463-2.

## Introduction

The number of infections with multidrug-resistant (MDR) Enterobacterales in humans has been increasing worldwide (Naghavi et al. 2024). The increase of pathogens, which are resistant to critically important and last resort antibiotics, such as carbapenems, intensifies the need for alternative treatment options. In the past, the antibiotic colistin (polymyxin E) was only used exceptionally as last resort in human medicine due to its nephrotoxic effect, and especially for specific treatments, such as local application or an aerosol for inhalation in patients with cystic fibrosis or *Pseudomonas* colonisation (Poirel et al. 2016). However, considering increasing MDR Gram-negative bacteria, colistin use has been resumed and is now one of the last resort options in human medicine, especially against carbapenemase-producing Enterobacterales (WHO 2024). In the past, colistin has long been approved and extensively used in European livestock since the 1950 s especially in pigs and poultry, mostly given orally as therapeutic group treatment against bacterial intestinal disorders (Ewers et al. 2022). It was assumed that resistance to colistin was exclusively associated with chromosomal mutations of genes involved in lipopolysaccharide (LPS) synthesis and modification like mutations in the *pmrCAB* operon, which are mostly described in *Escherichia coli* (Kempf et al. 2013; Rhouma et al. 2023). The publication of a novel, plasmid-mediated colistin resistance mechanism based on the activity of a lipid A phosphoethanolamine transferase named MCR-1 in early 2016 marked a turning point for veterinary medicine (Liu et al. 2016). The discovery of plasmid-mediated colistin resistance in humans and animals revealed that colistin resistance genes can theoretically be transferred from one bacterial species to another, representing a risk of transmission to human pathogens. Since the discovery of *mcr-1*, nine other variants (*mcr-2* to *mcr-10*) have been described (Hussein et al. 2021). In the wake of the renewed importance of colistin in human medicine, the worsening antibiotic resistance situation worldwide and findings on colistin resistance transmission, the World Health Organization (WHO) listed this antibiotic as highest priority critically important antimicrobial for human medicine, a category that also includes carbapenems (WHO 2024). In 2019, the European medical agency (EMA) placed colistin in category B “restrict”, that is, antibiotics to be used only for clinical infections in the absence of an alternative, clinically effective antibiotic in a lower category. This is intended to minimise the risk of developing colistin resistance in the veterinary sector (EMA 2019). Consequently, sales of colistin for use in animals has decreased between 2017 and 2022 by over 40% in Europe (ECDC, EFSA and EMA 2024). A similar decline occurred in Switzerland (approximately 92% since 2012), although it is still in use for several indications and animal species including livestock (FOH and FSVO 2024). Indeed, colistin remains essential for veterinary medicine in special indications like enterotoxemic and septic *E. coli* infections in pigs and poultry (ECDC, EFSA and EMA 2024). Given the reduced but continuing use of colistin, livestock could theoretically serve as a reservoir for colistin-resistant bacteria. As a result, colistin-resistant bacteria could be transmitted to humans through direct contact with faeces or, more likely, via fresh meat contaminated during the production process. As part of the European programme on antimicrobial resistance in zoonotic and indicator bacteria from humans, animals and food, implemented since 2015, caecal samples from livestock at slaughterhouses and fresh meat at retail are regularly tested for the presence of indicator*,* third-generation cephalosporin-resistant and carbapenem-resistant *E. coli*, but this does not include testing for colistin-resistant *E. coli* (EFSA and ECDC 2024).

Therefore, we screened Swiss livestock and fresh meat samples from Swiss retail stores, based on the random and representative sampling strategy used within the framework of the European programme on antimicrobial resistance in zoonotic and indicator bacteria from humans, animals and food, for the presence of colistin-resistant *E. coli* with a selective enrichment method. A whole genome sequencing (WGS)-based approach was used to determine the mechanisms underlying the colistin resistance and their potential association with genetic lineages in the different matrices.

## Materials and methods

### Sample collection

Caecal samples from pigs, cattle and poultry were collected in Swiss slaughterhouses by veterinarians within the framework of the European programme on antimicrobial resistance 2019 and 2020, applying random sampling within a stratified system (EFSA and ECDC 2024). Sampling was carried out evenly throughout the year in slaughterhouses to cover at least 60% of the annually slaughtered population of calves under one year, pigs and broilers. Hence, in 2019, this sampling included the seven largest cattle (C1-C3 and CP1-CP4) and pig (P1-P3 and CP1-CP4) slaughterhouses. In 2020, the sampling included the five largest broilers slaughterhouses (B1-B5). In total, 298 samples from slaughter calves under one year (one caecal content per slaughter batch each), 350 samples from pigs (one caecal content per slaughter batch each), and 805 samples from broilers (five pooled caecal samples per flock) were analysed.

Additionally, fresh, chilled and untreated prepacked meat samples were taken at retail level by Swiss cantonal laboratories within the framework of the European programme on antimicrobial resistance 2020 and 2023, applying random sampling within a stratified system. The applied sampling scheme considered each canton's population density and market shares of retailers. Sampling was carried out evenly throughout the year for chicken meat in 2020 and for the second half of 2023 for beef and pork. For chicken and beef, meat originating from Swiss livestock or from abroad were taken according to the proportion of imported and domestic meat on the market. In total, 295 chicken meat samples were analysed, 180 produced with Swiss chicken meat and 115 samples with meat from abroad (France, Germany, Hungary, Slovenia). The prepacked meat samples were produced by nine Swiss producers and eight European producers (France, Germany, Hungary, Slovenia). For beef, in total 164 samples were analysed, 136 produced with Swiss beef and 28 samples with meat from abroad (Argentina, Austria, Canada, Ireland, Italy, Lithuania, Paraguay, Uruguay). The prepacked meat samples were all produced by 25 different Swiss producers. As pork was not imported in significant quantities, all samples (n = 163) consisted of Swiss pork from 24 different Swiss producers.

### Isolation and identification of colistin-resistant *E. coli*

1 g ± 0.1 g of caecal content or 25 g ± 0.5 g of meat was transferred to 9 mL or 225 mL buffered peptone water (BPW, Axonlab AG, Baden, Switzerland) and mixed prior to aerobic incubation for 20 ± 2 h at 37 ± 1 °C. After shaking, 1 mL of the mixture was diluted in 9 mL of BPW containing 2 mg/L colistin and further incubated under aerobic conditions for 20 ± 2 h at 37 ± 1 °C. Then, 50 µL of the selective enrichment was spread onto CHROMID® Colistin R agar (BioMérieux, Marcy l‘Etoile, France) and incubated under aerobic conditions for 20 ± 2 h at 37 ± 1 °C. For quality control, colistin-resistant *E. coli* KP37 (*mcr-2* positive) (Ewers et al. 2022) and colistin-susceptible *E. coli* ATCC 25922 were used as controls*.* From the CHROMID® Colistin R agar, large, pink-coloured colonies suspected to be *E. coli* were subcultured on Tryptone Soya Agar containing 5% sheep blood (TSA-SB) (Thermo Scientific™, Pratteln, Switzerland). After 20 ± 4 h under aerobic incubation at 37 ± 1 °C, the species were identified by Matrix-Assisted Laser Desorption/Ionisation Time-of-Flight Mass Spectrometry (MALDI-TOF MS) using the direct transfer protocol according to the manufacturer's instructions (Biotyper 3.0, Bruker Daltonics GmbH, Bremen, Germany). Isolates confirmed as *E. coli* were cryopreserved at −80 °C in Trypticase-Soy Bouillon containing 30% of glycerine.

Potential induction of chromosomal mutations in *E. coli* leading to colistin resistance during incubation in colistin selective broth is described by Lee et al. (2015). Therefore, we conducted preliminary tests with the colistin-susceptible *E. coli* ATCC 25922 strain and ten colistin-susceptible *E. coli* field strains. The *E. coli* ATCC 25922 strain induces colistin resistance during overnight incubation in the selective enrichment in nine out of ten rounds. Two out of the ten field strains developed phenotypically detectable colistin resistance.

### Minimum inhibitory concentration determination

The isolates were re-cultivated from cryopreservation on TSA-SB under aerobic conditions for 21 ± 3 h at 37 ± 1 °C. Minimum inhibitory concentrations (MIC) were determined by broth microdilution in cation adjusted Mueller–Hinton broth (CAMHB) using EUVSEC (in 2019–2020) or EUVSEC3 (in 2023) Sensititre plates (Thermo Scientific™), following the ISO 20776–1 (2019) standard. These plates were incubated for 18 ± 2 h at 35 ± 1 °C. *E. coli* ATCC 25922 and *E. coli* KP37 used for quality control, showed MIC results in the acceptable ranges. MIC interpretation was based on epidemiological cut-off (ECOFF) values published by EUCAST (https://mic.eucast.org/). The ECOFF separates the wild-type population, which is assumed to have no acquired resistance mechanisms from the non-wild-type population with phenotypically detectable acquired resistance mechanisms (Kahlmeter and Turnidge 2022). Non-wild-type organisms are considered to show ‘microbiological’ resistance (as opposed to ‘clinical’ resistance based on clinical breakpoints). The following (tentative) (T)ECOFF values were used: ampicillin (8 mg/L), azithromycin (16 mg/L), cefotaxime (0.25 mg/L), ceftazidime (1 mg/L), chloramphenicol (16 mg/L), ciprofloxacin (0.06 mg/L), gentamicin (2 mg/L), meropenem (0.06 mg/L), nalidixic acid (8 mg/L), sulfamethoxazole (64 mg/L), tetracycline (8 mg/L), tigecycline (0.5 mg/L) and trimethoprim (2 mg/L). For colistin, the ECOFF of 2 mg/L is identical to the clinical breakpoint in brackets, therefore the term “colistin-resistant” was used.

All *E. coli* isolates which showed a colistin MIC > 2 mg/L on the EUVSEC or EUVSEC3 plates (test range from 1 to 16 mg/L) were phenotypically confirmed by the MICRONAUT MIC-Strip Colistin system (Bruker Daltonics GmbH) (test range from 0.06 to 64 mg/L) according to the manufacturer's instructions. Isolates with confirmed colistin MIC > 2 mg/L were interpreted as colistin-resistant and submitted to WGS for further analyses.

### Whole genome sequencing and in silico analysis

For the *E. coli* isolated in 2019 and 2020, DNA extraction, quality and quantity control, as well as the library preparation procedures were performed as described previously by Aebi et al. (2023) to obtain short reads with Illumina technology. For the isolates from 2023, the DNA extraction was performed with the QIAamp DNA Mini Kit (Qiagen, Hilden, Germany) and the library preparation and short read Illumina sequencing were performed by Microsynth AG, Balgach, Switzerland. The raw reads were submitted to the Sequence Read Archive (SRA) database of the National Center for Biotechnology Information (NCBI), with BioProject accession number PRJNA1034604 and BioSample accession numbers SAMN38061634—SAMN38061656 for the isolates from caecal content, and BioProject accession number PRJNA1308828 and BioSample accession numbers SAMN50714378—SAMN50714404 for the isolates from fresh meat (Table S1).

**Fig. 1:**
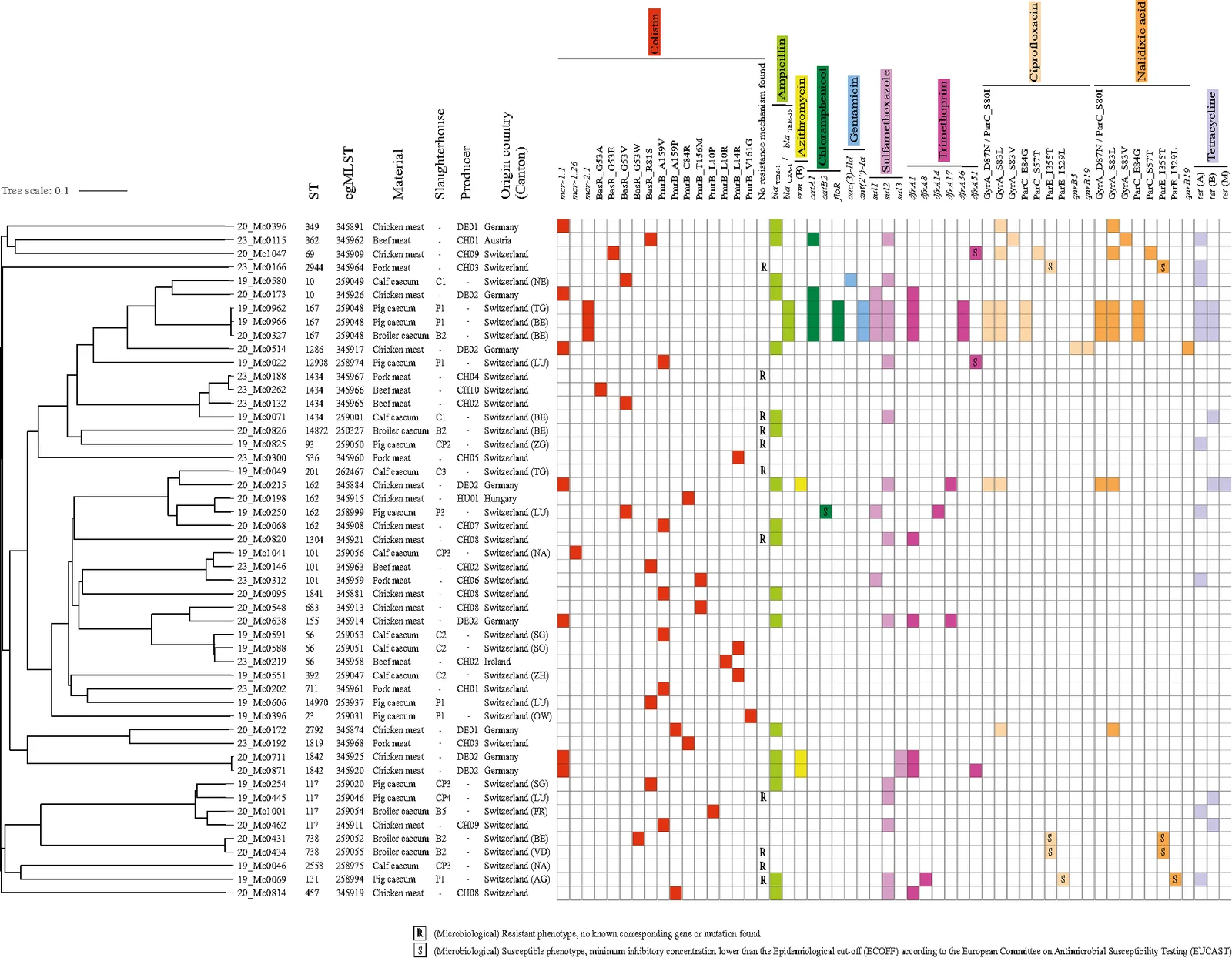
Phylogenetic tree based on cgMLST of the 50 colistin-resistant *Escherichia coli* found in this study, including 23 isolates found in caecal content from Swiss calves under one year, pigs and broilers at slaughter and 27 isolates found in beef, pork and chicken meat samples. The Neighbour-Joining Tree was created on Ridom SeqSphere^+^ (version 10.5.2, Ridom GmbH, Münster, Germany) and visualised with Phandango. The corresponding genetic characteristics and antimicrobial resistance mechanisms detected by WGS analysis are indicated with colours, coded per antibiotic type. ST, Sequence type; cgMLST, core genome ST. Slaughterhouses; C for calves (C1-3); P for pigs (P1, P3); CP for both calves and pigs (CP2-4), and B for broilers (B2, B5). Cantons of the farms: AG, Aargau; BE, Bern; FR, Fribourg; LU, Lucerne; NA, unknown; NE, Neuchâtel; OW, Obwalden; SG, Saint-Gallen; SO, Solothurn; TG, Thurgau; VD, Vaud; ZH, Zurich; ZG, Zug. Producers: meat produced in: Switzerland (CH01-10), Germany (DE01-02) and Hungary (HU01)

The raw reads were uploaded into Ridom SeqSphere^+^ (version 10.5.2, Ridom GmbH, Münster, Germany), where they underwent quality control (Table S1), were trimmed using Trimmomatic (v0.36) (Bolger et al. 2014) and assembled using SKESA (v. 2.4.0) (Ondov et al. 2016). The Ridom SeqSphere^+^ tool provided the following analysed data: species identification and contamination check (Mash Screen v.2.1) (Ondov et al. 2016), sequence type (ST) based on the Achtman *E. coli* multilocus sequence typing (MLST) scheme (MLST v2.0) (Larsen et al. 2012), antimicrobial resistance genes and point mutations (incorporated AMRFinderPlus (version 4.0.15, database version 2024–12.18.1)). For missing STs, new ones were attributed using Enterobase (EnteroBase (warwick.ac.uk)). The phylogenetic tree of the colistin-resistant *E. coli* core genome-based phylogeny as shown in Fig. 1 was created through Ridom SeqSphere^+^ with the incorporated Neighbor Joining Tree method (Saitou and Nei 1987), pairwise ignoring missing values, and visualised with Phandango v1.3.0 (Hadfield et al. 2018). Additionally, the core genome MLST (cgMLST) were analysed based on a 2520 allele scheme using Enterobase and used in Fig. 1. In parallel, the assemblies were uploaded into the ResFinder (v4.3.3, default parameters) provided by the Center for Genomic Epidemiology (CGE) (www.genomicepidemiology.org/) (Zankari et al. 2012) to strengthen completeness of antimicrobial resistance gene detection with the exception of colistin resistance. Note that for the detection of chromosomal mutations associated with colistin resistance, the AMRFinderPlus database included in Ridom SeqSphere^+^ was used since it includes 189 chromosomal mutations listed as associated with colistin resistance (https://www.ncbi.nlm.nih.gov/pathogens/) including the only three mutations (PmrA_S39I, PmrA_R81S and PmrB_V161G) provided in ResFinder. In two separate cases, different outcomes were observed between the AMRFinderPlus and ResFinder (*mcr-1.26* versus *mcr-1.1*, and *aac(3)-IId* versus *aac(3)-IIa,* respectively), leading to sequence analysis by read mapping using Geneious (www.geneious.com), which confirmed AMRFinder results. All phenotypic and genotypic results were compared and in case of discrepancies verified in duplicate.

### Statistics

The confidence intervals (CIs) were calculated using the Clopper-Pearson method on Ausvet Epitools Epidemiological Calculators (https://epitools.ausvet.com.au/ciproportion).

## Results

### Prevalence and characteristics of colistin-resistant *E. coli* from calves under one year, 2019

From 298 calves’ caecal samples, eight phenotypically colistin-resistant *E. coli* were identified, which represents a prevalence of 2.7% (95% CI 1.2–5.2%) (Table 1). They were isolated from calves slaughtered in four out of the seven Swiss slaughterhouses (Table 2).

**Table 1:** Prevalence of colistin-resistant *Escherichia coli* in caecal samples from Swiss calves under one year, fattening pigs and broilers, and in beef, pork and chicken meat samples

Material	Number of samples tested (n)	*E. coli* colistin resistant			*E. coli mcr*-positive		
		Number (n)	Prevalence (%)	95% CI	Number (n)	Prevalence (%)	*mcr*-genes
Calf caecum	298	8	2.7	1.2–5.2	1	0.3	*mcr-1.26*
Pig caecum	350	10	2.9	1.4–5.2	2	0.6	*mcr-2.1*
Broiler caecum	805	5	0.6	0.2–1.4	1	0.1	*mcr-2.1*
Beef	164	5	3.1	1.0–7.0	0	0	–
Pork	163	6	3.7	1.4–7.8	0	0	–
Chicken meat	295	16	5.4	3.1–8.7	7	2.4	*mcr-1.1*

**Table 2:** Prevalence of colistin-resistant *Escherichia coli* in caecal samples from Swiss calves, pigs and broilers taken at different slaughterhouses, and beef, pork and chicken meat producers

Material	Slaughterhouse/Producer	Number of samples tested (n)	Number of positive samples (n)	Prevalence (%)	95% CI
Calf caecum	C1	30	2	6.7	0.8–22.1
Calf caecum	C2	108	3	2.8	0.6–7.9
Calf caecum	C3	33	1	3.0	0.1–15.8
Calf caecum	CP3	67	2	3.0	0.4–10.4
Pig caecum	P1	102	6	5.9	2.2–12.4
Pig caecum	P3	6	1	16.7	0.4–64.1
Pig caecum	CP2	36	1	2.8	0.1–14.5
Pig caecum	CP3	104	1	1.0	0.0–5.2
Pig caecum	CP4	28	1	3.6	0.1–18.4
Broiler caecum	B2	382	4	1.1	0.3–2.7
Broiler caecum	B5	86	1	1.2	0.0–6.3
Beef	CH01	46	1	2.2	0.1–11.5
Beef	CH02	24	3	12.5	2.7–32.4
Beef	CH10	1	1	100.0	2.5–100
Pork	CH01	20	1	5.0	0.1–24.9
Pork	CH03	12	2	16.7	2.1–48.4
Pork	CH04	24	1	4.2	0.1–21.1
Pork	CH05	27	1	3.7	0.1–19.0
Pork	CH06	20	1	5.0	0.1–24.9
Chicken meat	CH07	1	1	100.0	2.5–100
Chicken meat	CH08	57	4	7.0	2.0–17.0
Chicken meat	CH09	34	2	5.9	0.7–19.7
Chicken meat	DE01	9	2	2.2	0.3–7.6
Chicken meat	DE02	14	6	42.9	17.7–71.1
Chicken meat	HU01	22	1	4.6	0.1–22.8

CI: confidence interval. Slaughterhouses: C for calves (C1-3); P for pigs (P1, P3); CP for both calves and pigs (CP2-4), and B for broilers (B2, B5). Producers: meat produced in Switzerland (CH01-10), in Germany (DE01-02) and in Hungary (HU01)

The eight isolates belonged to seven STs and eight cgSTs (Fig. 1). Two isolates belonged to ST56 and had distinct cgSTs with 185 loci difference. These two ST56 isolates were found in calves raised in different cantons, which were slaughtered the same day in the same slaughterhouse.

One isolate of ST101 contained the acquired *mcr-1.26* gene, four had chromosomal mutations (ST10, ST56 (n = 2), ST392), and three isolates (ST201, ST1434, 2558) exhibited no known resistance mechanism associated with colistin resistance (Fig. 1). Within these eight colistin-resistant *E. coli*, one ST101 and one ST1434 isolates showed additional phenotypic microbiological resistance to ampicillin (n = 2), gentamicin (n = 1), sulfamethoxazole (n = 2) and tetracycline (n = 2), for which corresponding resistance genes were identified (Fig. 1).

### Prevalence and characteristics of colistin-resistant *E. coli *from pigs, 2019

From 350 pigs’ caecal samples, ten phenotypically colistin-resistant *E. coli* were identified, which represents a prevalence of 2.9% (95% CI 1.4–5.2%) (Table 1). They were isolated from pigs slaughtered in five out of the seven Swiss slaughterhouses (Table 2).

The ten isolates belonged to eight STs and nine cgSTs (Fig. 1). Two isolates belonged to ST117 and had distinct cgSTs with 292 loci difference. These two ST117 isolates were found in pigs raised in different farms and slaughtered at different times and slaughterhouses. In contrast, two isolates belonged to ST167 and the same cgST, came from pigs raised in different farms, but slaughtered in the same slaughterhouse at the same time. One isolate (19_Mc0606) was identified as a new ST (ST14970).

The two ST167 isolates contained the acquired *mcr-2.1* gene*,* five had chromosomal mutations (ST23, ST117, ST162, ST12908, ST14970), and three isolates (ST93, ST117, ST131) exhibited no known resistance mechanism associated with colistin resistance (Fig. 1). Within these ten colistin-resistant *E. coli*, eight showed additional phenotypic microbiological resistance (ST93, ST117 (n = 2), ST131, ST162, ST167 (n = 2), ST12908) to ampicillin (n = 4), chloramphenicol (n = 2), ciprofloxacin and nalidixic acid (n = 2), gentamicin (n = 2), sulfamethoxazole (n = 7), tetracycline (n = 6) and trimethoprim (n = 4), for which corresponding resistance genes or point mutations were identified (Fig. 1). For two isolates (ST162, ST12908), additional resistance mechanisms (*catB2*, *dfrA51*) were detected although they showed MICs below the ECOFF for the corresponding antimicrobial (chloramphenicol, trimethoprim) (Fig. 1**)**. For the ST131 isolate showing microbiological susceptibility to ciprofloxacin and nalidixic acid, a single resistance mechanism (ParE_I529L) was detected, which is known not to be sufficient in *E. coli* to confer resistance to quinolones (Fig. 1) (Kalinen et al. 2025).

### Prevalence and characteristics of colistin-resistant *E. coli* from broilers, 2020

From 805 broilers caecal samples, five phenotypically colistin-resistant *E. coli* were identified, which represents a prevalence of 0.6% (95% CI 0.2–1.4%) (Table 1). They were isolated from broilers slaughtered in two out of the five Swiss slaughterhouses (Table 2).

The five isolates belonged to four STs and five cgSTs (Fig. 1). Two isolates belonged to ST738 and differed in the cgST by six loci. These two ST738 isolates were found in broilers raised in different farms but slaughtered at the same time and slaughterhouse. One isolate (20_Mc0826) was identified as a new ST (ST14872).

One ST167 isolate contained the acquired *mcr-2.1* gene, two had chromosomal mutations (ST117, ST738) and two isolates (ST738, ST14872) exhibited no known resistance mechanism associated with colistin resistance (Fig. 1). Within these five colistin-resistant *E. coli*, three (ST117, ST167 ST14872) showed additional phenotypic microbiological resistance to ampicillin (n = 2), chloramphenicol (n = 1), ciprofloxacin and nalidixic acid (n = 1), gentamicin (n = 1), sulfamethoxazole (n = 1), tetracycline (n = 2) and trimethoprim (n = 1), for which corresponding resistance genes or point mutations were identified (Fig. 1). For the two isolates belonging to ST738 and showing microbiological susceptibility to ciprofloxacin and nalidixic acid, a single resistance mechanism (ParE_I355T) was detected, which is known not to be sufficient in *E. coli* to confer resistance to quinolones (Fig. 1) (Kalinen et al. 2025).

### Prevalence and characteristics of colistin-resistant *E. coli* from beef, 2023

From 164 beef samples, five phenotypically colistin-resistant *E. coli* were identified, which represents an overall prevalence of 3.1% (95% CI 1.0–7.0%) (Table 1). These samples included 136 Swiss meat, from which three isolates were found (2.2% (95%CI 0.5–6.3%)) and 28 meat from abroad with two isolates detected (7.1% (95%CI 0.9–23.5%)). All five colistin-resistant *E. coli* were detected in beef prepacked by three out of the 25 Swiss producers. Apart from the producer CH10, from which the only sample taken tested was positive (100%), producer CH02 showing the second highest prevalence (12.5%) (Table 2).

The five isolates belonged to four STs and five cgSTs (Fig. 1). Two isolates belonged to ST1434 and had distinct cgSTs with 221 loci difference. These two ST1434 isolates were found in Swiss beef prepacked at two different Swiss producers at different time.

While no *mcr* genes were detected, chromosomal mutations associated with colistin resistance were detected for the five isolates (ST56, ST101, ST362, ST1434 (n = 2)) (Fig. 1). The ST362 isolate showed additional phenotypic microbiological resistance to ampicillin, chloramphenicol, ciprofloxacin and nalidixic acid, sulfamethoxazole and tetracycline, for which corresponding resistance genes or point mutations were identified (Fig. 1).

### Prevalence and characteristics of colistin-resistant *E. coli* from pork, 2023

From 163 Swiss pork samples, six phenotypically colistin-resistant *E. coli* were identified, which represents a prevalence of 3.7% (95% CI 1.4–7.8%) (Table 1). These samples were prepacked by five out of the 24 different Swiss producers (Table 2), which account together for 103 out of the 163 samples taken. The prevalence of colistin-resistant *E. coli* for these five Swiss producers (CH01, CH03 – CH06) was 5.8% (95% CI 2.2–12.3%).

The six isolates belonged to six different STs and cgSTs (Fig. 1). While no *mcr* genes were detected, four isolates had chromosomal mutations (ST101, ST536, ST711, ST1819), and two isolates (ST1434, ST2944) exhibited no known resistance mechanism associated with colistin-resistance (Fig. 1). Within these six colistin-resistant *E. coli,* one ST101 and one ST2944 isolates showed additional phenotypic microbiological resistance to sulfamethoxazole (n = 1) and tetracycline (n = 2), for which corresponding resistance genes were identified (Fig. 1). For the ST2944 isolate showing microbiological susceptibility to ciprofloxacin and nalidixic acid, a single resistance mechanism (ParE_I355T) was detected, which is known not to be sufficient in *E. coli* to confer resistance to quinolones (Fig. 1) (Kalinen et al. 2025).

### Prevalence and characteristics of colistin-resistant *E. coli* from chicken meat, 2020

From 295 chicken meat samples, 16 phenotypically colistin-resistant *E. coli* were identified, which represents an overall prevalence of 5.4% (95% CI 3.1–8.7%) (Table 1). These samples included 180 Swiss chicken meat samples, from which seven were positive (3.9% (95%CI 1.6–7.9%)), and 115 meat samples from abroad with eight positives from Germany and one from Hungary (7.8% (95%CI 3.6–14.3%)). The seven positive Swiss samples were prepacked by only three out of the nine Swiss producers (CH07 – CH09) (7.6%. (95% CI 3.1–15.1%)). All eight positive samples produced with German meat were prepacked by German producers (DE01, DE02) (34.8% (95% CI 16.4–57.3%)).

The 16 colistin-resistant *E. coli* belonged to 13 STs and 16 cgSTs (Fig. 1). Three isolates belonged to ST162 and had distinct cgSTs with 563, 627 and 667 loci difference between each other. These three ST162 isolates were found in Swiss, Hungarian and German chicken meat prepacked by different producers (CH07, HU01, DE02 respectively). In contrast, two isolates belonged to ST1842 isolates and differed in the cgST by two loci. Both isolates were found in German meat prepacked at different time by the same German producer (DE02), which accounted for another four positive samples with different STs.

Seven isolates (ST10, ST155, ST162, ST349, ST1286, ST1842 (n = 2)) contained the acquired *mcr-1.1* gene, all of which isolated from German chicken meat and producers (DE01, DE02), eight isolates (ST69, ST117, ST162 (n = 2), ST457, ST683, ST1841, ST2792) had chromosomal mutations, and one isolate (ST1304) exhibited no known resistance mechanism associated with colistin resistance (Fig. 1). Within these 16 colistin-resistant *E. coli*, 14 isolates (ST10, ST69, ST117, ST155, ST162 (n = 2), ST349, ST457, ST1286, ST1304, ST1841, ST1842 (n = 2), ST2792) showed additional phenotypic microbiological resistance to ampicillin (n = 12), azithromycin (n = 3), chloramphenicol (n = 1), ciprofloxacin and nalidixic acid (n = 5), sulfamethoxazole (n = 8), tetracycline (n = 2), and trimethoprim (n = 7), for which corresponding resistance genes or point mutations were identified (Fig. 1). For the ST69 isolate, an additional resistance mechanism (*dfrA51*) was detected although it showed MICs below the ECOFF the corresponding antimicrobial (trimethoprim) (Fig. 1).

## Discussion

By applying a selective enrichment and bacterial culturing method, the present study revealed an overall low prevalence of colistin resistance in *E. coli* from Swiss livestock at slaughterhouse level, with the lowest prevalence detected in broilers (0.6%), followed by calves (2.7%) and pigs (2.9%). These generally low prevalences might be even lower, if one considers that chromosomal mutations may have been induced during overnight incubation in the selective enrichment medium. For *mcr*-mediated colistin resistance, the prevalences were even lower (broilers 0.1%, calves 0.3%, pigs 0.6%). These results are slightly, but not much higher compared to findings for indicator *E. coli* in Swiss livestock performed in the framework of the European programme on antimicrobial resistance in zoonotic and indicator bacteria from humans, animals and food based on non-selective methods for colistin resistance. From 2019 to 2023, approximately 200 indicator *E. coli* from caecal samples of slaughtered calves, pigs and broilers – isolated by direct plating on Mac Conkey agar—were phenotypically analysed each year for colistin resistance by MIC testing, but no colistin-resistant isolate was detected (0.0% (95% CI 0.0–1.8%)) (FOH and FSVO 2020, 2022, 2024). The same was observed for third-generation cephalosporin-resistant *E. coli,* isolated from caecal samples by non-selective enrichment and selective plating, which were all phenotypically susceptible to colistin. Similarly, in most European Union countries, resistance to colistin is rarely detected in indicator *E. coli* isolated from livestock by non-selective methods as part of the European harmonised monitoring programme on antimicrobial resistance (EFSA and ECDC 2024). Most countries (62–82%) reported either low numbers of colistin-resistant *E. coli* or none in any tested livestock population, including turkeys. However, moderate percentages of colistin resistance were detected in indicator *E. coli* from turkeys in Poland and Portugal between 2021 and 2022 (19.1% and 11.2% respectively), which might indicate that the European harmonised, non-selective method for indicator *E. coli* will detect relevant levels of colistin resistance if present in specific animal population. In contrast to caecal samples, indicator *E. coli* from meat taken at retail is not included in the European programme on antimicrobial resistance (EFSA and ECDC 2024), thus providing no data regarding prevalence. The 27 colistin-resistant *E. coli* isolated in meat in this study have been detected solely using the colistin-selective method and not through the already implemented detection of third-generation cephalosporin and carbapenem resistant *E. coli,* for which colistin resistance is additionally assessed phenotypically*.* For Swiss beef and Swiss pork, the prevalence of colistin-resistant *E. coli* isolated using the colistin-selective enrichment and bacterial culturing method was within the same ranges as those found in Swiss caecal samples from calves and pigs at slaughterhouse, with 3.1% in beef (2.2% in domestic meat, but 7.1% in foreign meat) and 3.7% in pork. No *mcr* genes were detected among these samples, which were all prepacked by Swiss producers. However, it must be taken into consideration that some of the chromosomal mutations detected might have been induced by the selective overnight incubation. In contrast to beef and pork, importation of chicken meat into Switzerland is due to a domestic production which does not cover the market demand. Overall, the prevalence of colistin-resistant *E. coli* was higher in chicken meat than in Swiss broiler caecal samples (5.4% and 0.6%, respectively), due to a higher percentage of colistin-resistant *E. coli* found in imported meat than in Swiss meat. In imported chicken meat, the prevalence was 7.8%, with six of the seven *mcr*-containing *E. coli* isolated from German meat produced by one producer (DE02). The prevalence was also higher in Swiss chicken meat with 3.9% than in Swiss broiler caecal samples (0.6%), suggesting that contamination occurs at meat processing level as already observed previously for third-generation cephalosporin resistant *E. coli* (Vogt et al. 2014). Notably, seven positive Swiss chicken meat originated from only three Swiss producers out of nine, and four solely by the manufacturer CH08. Although colistin resistance in *E. coli* from Swiss meat was only associated with chromosomal mutations in our study and might therefore be partially induced by the overnight incubation, the presence of *mcr-1* was already observed in third-generation cephalosporin-resistant *E. coli* isolated from fresh Swiss chicken meat in 2016 (Donà et al. 2017). The *mcr-1* was also detected in one of 122 third-generation cephalosporin-resistant *E. coli* in Portuguese broilers raised in intensive systems, indicating that the presence of plasmid-mediated in third-generation cephalosporin-resistant *E. coli* is rather low (Ferreira et al. 2022). In addition, Lima et al. (2022) showed in vitro that a loss of *mcr-1* bearing plasmids in *E. coli* is not directly correlated with the absence of colistin, which is an important finding for understanding the development of colistin resistance in livestock, particularly given the restrictions on the use of colistin resulting from its classification as a category B antibiotic.

Monitoring of colistin resistance should be extended to fresh meat to provide competent authorities with more specific data on the transmission risk for colistin-resistant *E. coli* to humans via the food chain. In this regards, Perrin-Guyomard et al. (2022) have already evaluated a selective isolation protocol for the detection of *mcr-*positive *E. coli* and *Salmonella* in the framework of the European harmonised monitoring programme. However, the method used is exclusively based on a multiplex PCR for the detection of known *mcr* genes. With such a method, new emerging colistin resistance mechanisms as well as mutations will be missed, and the actual prevalence will be biased. Our study was based on an approach using selective bacterial culturing, which permitted the isolation of *E. coli* which contain known and unknown *mcr* genes and chromosomal mutations associated with colistin resistance. The use of an overnight incubation in broth with 2 mg/L colistin may induce for chromosomal mutations as shown by Lee et al. (2015) and was confirmed in our preliminary assays, thus overestimating the prevalence of colistin resistance associated with chromosomal mutations in *E. coli* in this study. However, our prevalences show that an induction rate of 20 per cent – based on preliminary tests with ten field strains – cannot be applied to the selective enrichment of caecal and meat samples, as the prevalences observed are much lower than the calculated prevalences based on the assumption of 20 per cent induction rate. Nevertheless, it is necessary to develop a selective detection procedure, which prevent induction of chromosomal mutations.

The WGS analysis of the 50 phenotypically colistin-resistant *E. coli* strains showed that besides an overall low prevalence of colistin resistance, most detected resistance mechanisms were associated with chromosomal mutations possibly induced by the selective overnight incubation (n = 28) and not through the acquisition of known *mcr* genes (n = 11). No known resistance mechanism associated with phenotypic colistin resistance could be identified in 11 isolates. Chromosomal mutations in regions coding for proteins responsible for modifications of lipid A occur naturally and a mutation responsible for colistin resistance is difficult to identify because of the great variety of involved enzymes (Raetz et al. 2007). Therefore, we reported known chromosomal mutations and did not analyse systematically for other potential chromosomal mutations. Since the discovery of the *mcr-1* gene in 2016, research was focused on the detection of plasmid-mediated *mcr* genes conferring colistin resistance, as such genes could easily be exchanged between the different bacterial species of Enterobacterales. The genetic location of *mcr* genes of strains from our study was not further investigated as it was beyond the scope of this study. Analysis of complete genome assemblies obtained from long read sequencing would permit genomic localization of *mcr* genes and serve as baseline for comparative epidemiological studies of *mcr*-containing mobile genetic elements. In this regard, a previous study revealed that plasmid-mediated *mcr-1* gene in colistin-resistant *E. coli* from humans in Switzerland and imported raw chicken meat may have a common origin emphasizing the need for WGS-based surveillance (Donà et al. 2017).

In addition to colistin resistance, 20 out of the 50 isolated *E. coli* were susceptible to all other antimicrobials tested. The remaining 30 colistin-resistant *E. coli* showed phenotypic resistance to commonly used, first line antimicrobials for livestock, like ampicillin, tetracyclines, sulfonamides, trimethoprim and phenicols. Azithromycin resistance was observed in three *erm*(B)-containing *E. coli* from chicken meat, confirming previously findings, that *erm* genes lead to elevated azithromycin MIC (Ivanova et al. 2024). In three strains, a gene was detected (*catB2*, twice *dfrA51*), while no phenotypic resistance to chloramphenicol or trimethoprim, respectively, was observed. Absence of a phenotype in the presence of a corresponding resistance gene is a known phenomenon, which might be due to e.g. truncations, mutations and down regulation (Yee et al. 2021).

Epidemiological WGS analysis revealed a high diversity of the 50 colistin-resistant *E. coli* as they belonged to 32 STs and 48 cgSTs. The discriminatory power of cgMLST in comparison to traditional MLST was observed with isolates belonging to the same ST but differing in cgSTs with > 150 loci, indicating no epidemiological relatedness between these isolates. In three instances, the isolates belonging to the same ST had less than 10 loci differences (ST167, ST738, ST1842) in cgST, which could indicate that genetically related strains of specific ST may be more adapted for persistence and dissemination in animals, slaughterhouses or production sites. However, no environmental sampling was carried out to proof this hypothesis.

The low prevalence of colistin-resistant *E. coli* may result from the constantly decreasing usage of colistin in food-producing animals in Switzerland (FOH and FSVO 2024). It should also be noted that samples were taken from animals only at slaughterhouse level and not at an earlier stage in the production process, when treatment with colistin is more likely. A study in the Netherlands and Belgium found a positive correlation between colistin resistance in Enterobacterales and prior colistin use, leading to high colistin resistance (> 50%) in Enterobacterales in times when colistin was used during and in between time points of sampling (De Koster et al. 2024). However, they did not observe any inter-host transmission between livestock animals and human, nor did other studies.

On the European level, no data on colistin resistance in human pathogens are available, but the use of colistin in human medicine increased from 2014 to 2021 (ECDC, EFSA and EMA 2024). The use of colistin in human medicine is strongly linked with the occurrence of carbapenemase-producing pathogens, which differs substantially between the European countries (EFSA and ECDC 2024). It is foreseeable that the prevalence of colistin resistance in *E. coli* and other Enterobacterales from human will increase in the future due to the increased use of this last resort antimicrobial in human medicine.

## Conclusions

Our study reveals that despite low occurrence of colistin-resistant *E. coli* in domestic livestock, fresh meat, especially poultry meat, might be contaminated with colistin-resistant *E. coli.* But, until now, detection of phenotypically colistin-resistant *E. coli* is not implemented in the European harmonised monitoring programme on antimicrobial resistance in zoonotic and indicator bacteria from humans, animals and food. Applying an approach using selective bacterial culturing is needed to include all phenotypically colistin-resistant *E. coli,* irrespective of their underlying molecular mechanisms. However, further research is needed to develop an optimised method that prevents the induction of chromosomal mutations in bacteria during selective enrichment, which might overestimate detected prevalences. WGS-based analysis of phenotypically colistin-resistant *E. coli* is necessary to identify the molecular mechanisms and determine role of mobile genetic elements in the dissemination of colistin resistance genes in both human and animals, thus providing data for One Health surveillance and epidemiology.

## Supplementary Information

Below is the link to the electronic supplementary material.

Supplementary file1 (XLSX 15 KB)

## Data Availability

No datasets were generated or analysed during the current study.
